# Biomechanical properties of 3D-printed bone scaffolds are improved by treatment with CRFP

**DOI:** 10.1186/s13018-017-0700-2

**Published:** 2017-12-22

**Authors:** Carlos G. Helguero, Vamiq M. Mustahsan, Sunjit Parmar, Sahana Pentyala, John P. Pfail, Imin Kao, David E. Komatsu, Srinivas Pentyala

**Affiliations:** 1 Department of Mechanical Engineering, Stony Brook University, Stony Brook, NY USA; 2 Department of Orthopedics, Stony Brook Medical Center, Stony Brook, NY USA; 3 Department of Anesthesiology, Stony Brook Medical Center, Stony Brook, NY USA; 4 Facultad de Ingeniería en Mecánica y Ciencias de la Producción, Escuela Superior Politécnica del Litoral, ESPOL, Guayaquil, Ecuador

## Abstract

**Background:**

One of the major challenges in orthopedics is to develop implants that overcome current postoperative problems such as osteointegration, proper load bearing, and stress shielding. Current implant techniques such as allografts or endoprostheses never reach full bone integration, and the risk of fracture due to stress shielding is a major concern. To overcome this, a novel technique of reverse engineering to create artificial scaffolds was designed and tested. The purpose of the study is to create a new generation of implants that are both biocompatible and biomimetic.

**Methods:**

3D-printed scaffolds based on physiological trabecular bone patterning were printed. MC3T3 cells were cultured on these scaffolds in osteogenic media, with and without the addition of Calcitonin Receptor Fragment Peptide (CRFP) in order to assess bone formation on the surfaces of the scaffolds. Integrity of these cell-seeded bone-coated scaffolds was tested for their mechanical strength.

**Results:**

The results show that cellular proliferation and bone matrix formation are both supported by our 3D-printed scaffolds. The mechanical strength of the scaffolds was enhanced by trabecular patterning in the order of 20% for compression strength and 60% for compressive modulus. Furthermore, cell-seeded trabecular scaffolds modulus increased fourfold when treated with CRFP.

**Conclusion:**

Upon mineralization, the cell-seeded trabecular implants treated with osteo-inductive agents and pretreated with CRFP showed a significant increase in the compressive modulus. This work will lead to creating 3D structures that can be used in the replacement of not only bone segments, but entire bones.

## Background

There is a significant interest in the medical community to create artificial bones that can mimic the natural bone. Synthetic bone scaffolds have been used to replace diminished bone stock, aid in fracture repair, and assist in the integration of orthopedic implants to the native bone [1]. Implantable bone technology has a tremendous potential in health care. It is estimated that current global orthopedic implant market is $34.9 billion, and the market is expected to grow at a rate of 4.9% over the next 5 years. The aging population and associated increased risk of osteoporosis, osteoarthritis, bone injuries, and obesity are significant contributors to the need for orthopedic implants.

Current 3D printing bone technologies can create either hard inert bone structures (based on primary scaffolds) that are structurally compatible but still functionally inert or fragile soft structures that have osteoconductive properties but are extremely weak in structure. However, the best bone scaffolds should be able to withstand heavy loads and, at the same time, allow for osteoconductivity [2]. Several studies tested the biomimicry and structural strength of different printing materials. However, all attempts were unsuccessful in finding the perfect material [3]. Low mechanical strength is a major challenge in porous scaffolds and is primarily controlled by pore volume. This is also true for one-dimensional (1D) and 3D-printed scaffolds and limits their use to only non-load bearing and low-load bearing applications. As such, we propose to create a load bearing artificial bone using acrylonitrile butadiene styrene (ABS) polymer. ABS-M30i is a biocompatible 3D printing material that can be used to print surgical models directly from CAD data.

Currently, artificial bone is being used to treat segmental defects, particularly for trauma and oncology patients. Several materials are available to treat segmental defects, but all are encumbered by significant problems that limit their efficacy [4]. While numerous materials are clinically available, or under development to treat segmental defects, each carries a unique constellation of benefits and drawbacks that forces the surgeon to compromise some aspects of patient care upon selection. They can resorb too quickly, are prone to prolonged drainage issues, can be hard to keep in place, and despite offering some structural support in compression, are generally brittle [5]. To overcome these difficulties and to create a biomimetic and biocompatible artificial bone, an attempt was made to create implantable scaffolds seeded with bone producing cells that are enhanced by osteogenic agents.

We developed computer algorithms that are capable of scanning protein sequence databases to yield protein segments that are highly enriched in precursors of known biopeptides. One of the hits, a peptide with the sequence KRQWAQFKIQWNQRWGRR, was mapped to the intracellular C-terminal region of the human calcitonin receptor. This region of the receptor has been shown to be its G-protein interaction site [6], and it is highly conserved across species. Moreover, this peptide contains consensus sequences for processing and also C-terminus amidation that results in the 12-amino acid long peptide, WAQFKIQWNQRW-amide (CRFP) [7]. CRFP was found to be bioactive as well as osteogenic by its ability to enhance bone matrix production by osteoblasts and by its ability to transform stem cells into bone-producing cells. CRFP was also found to be skeletally bioactive in vivo by strengthening bones in osteoporotic animals [8], and hence, CRFP along with other osteogenic agents was used in this study to increase the bone matrix production on scaffolds.

The purpose of this study is to create artificial bone that is both biomimetic and biocompatible using structural designs obtained from μCT of bones for creating bone scaffolds and strengthen these scaffolds by seeding them with bone-producing cells.

## Methods

### Design process for 3D-printed scaffolds using trabecular bone patterning

**Fig. 1:**
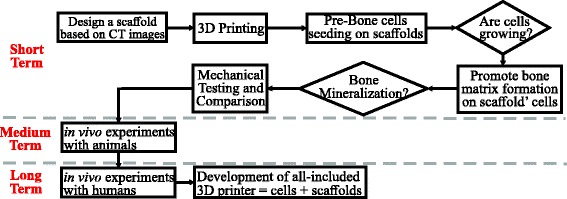
Flowchart for biomimetic implants project. During the first phase, designing scaffolds based on the trabecular pattern of the bone was assessed. Later, cell cultural response of scaffolds and mineralization was achieved. Mechanical compression tests were conducted to compare seeded scaffolds properties with real bone. Future work will include in vivo testing in animal models and human patient trials. The final goal is to develop an all-in-one equipment for bioprinting cell-seeded implants

A reverse engineering method was used to design 3D-printed ABS scaffolds (Fig. 1). An L5 vertebrae from a skeletally mature, male Sprague Dawley rat was isolated and scanned by micro-computed tomography (μCT40, Scanco Medical) at an isotropic voxel resolution of 18 um. The output was extracted to a series of DICOM files, each file describing a 2D cross-section representing of the site being imaged. Using InVesalius (Renato Archer Information Technology Center, Brazil), a medical imaging reconstruction software that takes the DICOM files from the CT scan and creates a three-dimensional (3D) surface representation, 3D image was exported into a stereolitography (STL) format, a standard among CAD software and 3D printing. To reduce the noise from the DICOM files, Geomagic (3D-systems, NC) was used as a STL image processing software. Once the noise of the STL file was completely reduced, a Geomagic algorithm was used to export the output as a CAD file. This file was uploaded to Solidworks (Dassault Systèmes, MA) (solid modeling CAD engineering software which is used for feature extraction to replicate the bone’s trabeculae pattern over scaffold’s surface) to export it as STL file which was used as an output to the 3D printer. The scaffolds were 3D printed using fused deposition modeling (FDM) process in a CubePro (3D Systems, USA) 3D printer. In order to successfully extract the trabecular pattern of the bone so that it matched our 3D printer resolution, several reiterations were made so as to resemble the actual bone, and the best structurally biomimetic scaffold was selected for our studies.

### Cellular growth on 3D-printed ABS scaffolds

MC3T3 cells were used to seed on scaffolds as they are commonly used to study in vitro osteoblast differentiation [8]. The printed scaffolds were washed for 24 h with deionized water and sterilized in ethanol also, for 24 h. After that, scaffolds were air-dried in a cell culture hood. Each scaffold was attached to the bottom of a cell culture plate well using sterilized vacuum grease. The scaffolds were pretreated with CRFP (15 uM) for 24 h and air-dried under sterile conditions. MC3T3 cells at a density of 1 × 10^3^ cells per chamber were seeded onto the scaffolds and cultured in media (DMEM supplemented with 10% fetal bovine serum and 1% penicillin/streptomycin). Media was changed every 3 days till the cells reached 70% confluency.

### Matrix deposition on 3D-printed scaffolds

Osteogenesis was induced and assessed as previously described. Glycerol phosphate (G6P) and ascorbic acid are well known osteogenic agents [9, 10]. It is also of interest to evaluate CRFP in these scaffolds as an osteogenic agent before seeding cells. Osteogenic reagents were added to each well as described earlier [8]. Confluent cells treated with osteogenic inducers were cultured for 21 days. Media were changed accordingly every 5 days.

### Evaluating cellular growth and bone matrix deposition

To evaluate the growth of cells on ABS scaffolds, scanning electron microscopy, fluorescence microscopy, and bright field microscopy were used. Cell-seeded and cultured scaffolds were fixed in 4% formaldehyde (Electron Microscopy Services) for 30 mins at room temperature. Two distinct staining protocols were used to assess cellular growth and osteogenesis. For cellular growth, scaffolds were double stained with Alexa 568-Phalloidin (Sigma-Aldrich) and DAPI (Sigma-Aldrich) to visualize actin and nuclei, respectively. To evaluate bone matrix deposition, 2% Alizarin red (Lifeline Cell Technologies) was used to stain calcium deposits for 20 mins at room temperature to evaluate mineralization process and bone matrix formation. Scaffolds were imaged with confocal microscopy (Zeiss LSM-510) and with a scanning electron microscope (ISI-SX30 SEM).

### Mechanical properties of biomimetic scaffolds

**Fig. 2:**
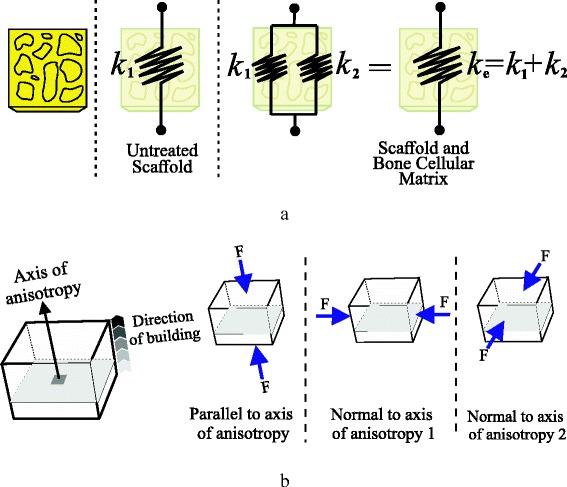
**a** Simplified mechanical model of springs to calculate stiffness of scaffolds. Untreated specimens will report only material’s stiffness *k*_1._ After treatment, scaffolds will increase their resistance as they also host for bone cellular matrix stiffness *k*_2_. This model of springs will allow us to calculate an equivalent-stiffness parameter *k*_e_. **b** Testing anisotropy of 3D-printed scaffolds. Different axes of compression are tested as 3D printing technology introduces anisotropy characteristics to scaffolds since they are being manufactured layer by layer

A mechanical model was used to obtain the stiffness, or modulus, of the specimens (Fig. 2). Briefly, a non-treated scaffold has only the stiffness of its material (in this case ABS), and it is denoted as *k*_1_. When the scaffolds are treated and mineralization is induced, the scaffolds now carry not only the ABS stiffness but also the stiffness of the formed cellular matrix *k*_2_. Mechanical testing cannot distinguish between these two stiffnesses, as it will only allow us to measure the equivalent stiffness of the hybrid, i.e., *k*_e_. A straightforward mechanical model will allow us to calculate *k*_e_ as follows:

1 $$ {k}_{\mathrm{e}}={k}_1+{k}_2 $$

In this study, the anisotropy of the scaffolds was tested Eq. 1. Three different directions of compression were tested and compared (Fig. 2).

**Fig. 3:**
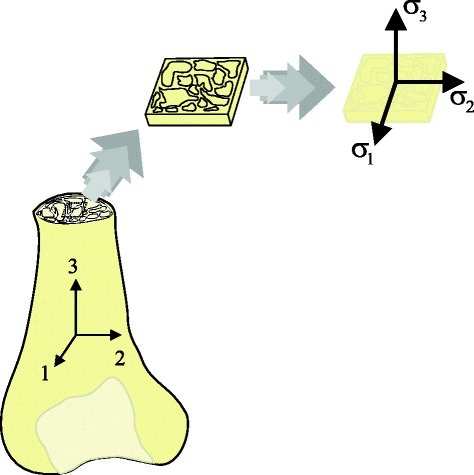
Relationship between the bone and scaffold axis of compression with respect to trabecular feature. Scaffold trabecular design accounts for the natural position of the bone and, therefore, its longitudinal axis for load bearing. Experimental results showed evidence that by accounting for this position, the mechanical strength of the scaffold will increase in that same axis

Also, not only the manufacturing process of the scaffolds contributes to their anisotropy, but also trabecular pattern itself adds this characteristic to the specimen as bone structure is indeed anisotropic. Bone structures perform better under compression rather than tension. However, the direction of the compression is also important. In previous statistical studies [11], the following inequality was noted, according to axis described in Fig. 3:

2 $$ {\sigma}_1\le {\sigma}_2<{\sigma}_3 $$

According to Eq. 2, the human bone performs better under compression but only if the axis of that compression is parallel to the longitudinal axis of the bone (Fig. 3). In our case, the trabecular feature of the bone was extracted from the natural anatomical position.

To carry out all these comparisons, the international standard, ISO 604 [12], was followed, which is a standard for determination of compressive properties of plastics. We chose this standard based on the core material of our scaffolds. As stated before, two main comparison tests were performed in the experiments using this standard on scaffolds seeded with cells with or without osteogenic agent treatment:

1. Flat scaffolds vs. trabecular scaffolds (non-treated specimens)
2. Treated vs. non-treated trabecular scaffolds

Two different dimensions of specimens were considered (Table 1). Type A models were used to calculate the compressive modulus of scaffolds, and type B models allowed us to obtain the compression strength. Table 2 and Fig. 2 detail the list of the number of specimens tested according to ISO 604 specifications and the specified directions of compressions.

**Table 1:** Measures of scaffolds used in ISO 604 compression test. All values in mm

Type	Measurement	Length	Width	Thickness
A	Modulus	50 ± 2	10 ± 0.2	4 ± 0.2
B	Strength	10 ± 2	10 ± 0.2	4 ± 0.2

**Table 2:** Number of specimens per anisotropy testing approach. Refer to Fig. 2 for axis labeling

Type	Quantity	Configuration	Anisotropy approach
A	5	Flat	Parallel to axis
A	5	Trabecular	Parallel to axis
A	5	Flat	Normal to axis 1
A	5	Trabecular	Normal to axis 1
A	5	Flat	Normal to axis 2
A	5	Trabecular	Normal to axis 2
B	5	Flat	Parallel to axis
B	5	Trabecular	Parallel to axis
B	5	Flat	Normal to axis 1
B	5	Trabecular	Normal to axis 1
B	5	Flat	Normal to axis 2
B	5	Trabecular	Normal to axis 2

An MTEST Quattro (Admet, USA) compression testing system was used in concordance with ISO 604 to carry out scaffolds testing. Specimens were tested in unconstrained compression by positioning them in the center of two stainless steel plates. Surface finishing of the specimens was enhanced prior to experiments using sandpaper in order to eliminate sharp edges and loose filaments formed due to 3D printing process. All environmental conditions established by ISO 604 were accomplished, stainless steel plate parallelism was inspected and corrected before any run, and every specimen was placed in the same position for any test to avoid discrepancies in results.

The speed for the tests varies depending on the outcome parameter and the dimension of the specimens. For our scaffolds, the following speeds were used as stated in ISO 604:

1. 1 mm/min for modulus measurement
2. 5 mm/min for strength measurement. This speed varies depending on the behavior of the material under compression [12]. Since ABS yields, ISO 604 requires 5 mm/min.

To evaluate biomechanical properties of cell-seeded scaffolds, compression strength (*σ*_M_) and compressive modulus (E_C_) were measured:

- Compression strength (*σ*_M_): maximum compressive stress sustained by the test specimen during a compressive test
- Compressive modulus (E_C_): ratio of the stress difference (*σ*_2_ − *σ*_1_) to the corresponding strain difference values (*ε*_2_ = 0.0025 minus *ε*_2_ = 0.005)

3 $$ {E}_{\mathrm{c}}=\frac{\sigma_2-{\sigma}_1}{\varepsilon_2-{\varepsilon}_1} $$

Average values for each set of five tests were taken to construct stress vs. strain curves based on Eq. 3.

## Results

### Cellular growth on ABS scaffolds

**Fig. 4:**
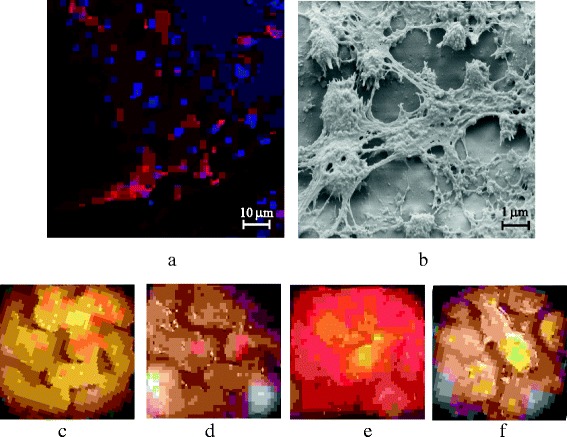
Microscopy results over treated scaffolds: **a** laser scanning confocal images of osteoblasts growing on 3D-printed scaffolds. Cells stained with Alexa Phalloidin and DAPI, **b** scanning electron microscope image of cells growing on 3D-printed scaffolds. Mineralization results using Alizarin red stain: **c** control (no reagents), **d** G6P + ascorbic acid, **e** CRFP, and **f** G6P + ascorbic acid + CRFP

Incubating the seeded scaffolds under osteogenic conditions showed osteoblasts promoting matrix formation and mineralization, as evidenced by Alizarin red staining in 3D reconstructed confocal z-sections images of cells grown on scaffolds (Fig. 4a). In addition, SEM imaging shows cells attached to the surfaces of the scaffolds (Fig. 4b).

### Biomechanics of osteogenic agents-treated cell scaffolds

Values for *σ*_M_ and E_C_ were shown in Table 3. For axis 1 and axis 2, flat scaffolds reported greater compressive strength and modulus; however, for axis 3, trabecular scaffolds exceeded flat specimens in the order of 20% for *σ*_M_ and 60% for E_C_.

**Table 3:** Compression test results: flat vs. trabecular scaffolds

	Axis 1		Axis 2		Axis 3	
	Flat	Trabecular	Flat	Trabecular	Flat	Trabecular
*σ*_M_	35.15	29.19	35.57	30.77	55.93	69.88
E_C_	242.96	166.46	236.99	188.69	133.60	336.65

Average values for *σ*_M_ and E_C_ for each axis of compression. Values in MPa

**Fig. 5:**
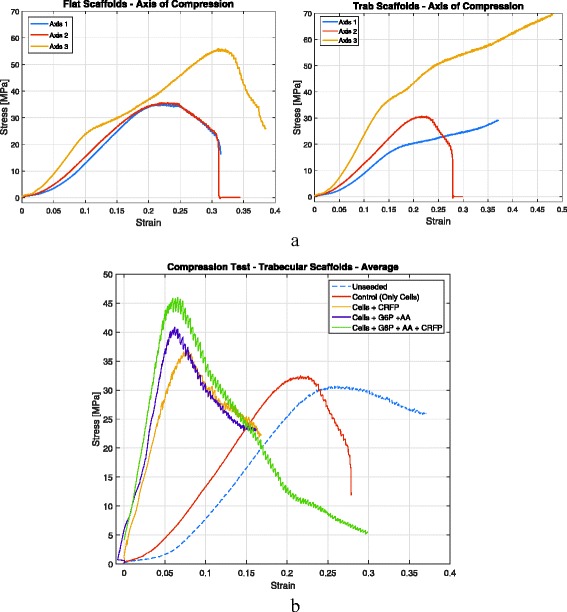
Compression test results: **a** for different axis of compression for either flat or trabecular scaffolds, showing that axis 3 of compression reports greater mechanical strength as it is the same axis for bone’s load bearing in natural position, (**b**) compression test results: treated vs. untreated scaffolds, showing that after inducing for mineralization, scaffolds increase both their compression strength and stiffness. Scaffolds treated with CRFP in addition to traditional osteogenic agents reported greater strength and stiffness

Another way to compare the results is from the point of view of axis of compression, grouping only flat scaffolds or trabecular scaffolds (Fig. 5a). Equation 2 shows that the compressive strength in axis 3 is greater than axis 2, which is also greater than axis 1.

The contribution of cellular bone matrix for compression strength and modulus was also investigated (Fig. 5b). Only trabecular scaffolds were compressed in direction 2. Four samples were tested: (1) unseeded, or untreated, scaffolds, which are the original specimens without any biological treatment or cell seeding;(2) seeded scaffolds, specimens with cells where mineralization was not induced; (3) seeded with osteogenic, specimens with cells induced for mineralization by using osteogenic reagents (G6P and ascorbic acid); and (4) seeded with CRFP, which are specimens with cells induced for mineralization by using CRFP.

Upon mineralization, the trabecular scaffolds showed an increase in the compressive modulus. Compressive strength also increased, but not as profoundly as E_C_. Table 4 shows average values for each set of five compression tests recommended by ISO 604. Treating seeded scaffolds with CRFP significantly increased the compressive modulus compared to unseeded controls.

**Table 4:** Compression test results: treated vs. untreated scaffolds. Values in MPa

	Unseeded	Control (only cells)	Cells + CRFP	Cells + G6P + ascorbic acid	Cells + G6P + ascorbic acid + CRFP
*σ*_M_	30.76	32.46	36.75	40.88	45.98
E_C_	169.46	199.44	560.69	737.10	860.02

## Discussion

Recent advances in 3D printing have enabled biocompatible materials to be applied to regenerative medicine in order to address the growing need for tissues and organs, including the bone. The aging population, with its concomitant increased risk of osteoporosis, osteoarthritis, bone injuries, and obesity, are significant contributors to orthopedic implant failure. Due to its ability to be tailored for patient-specific needs, the demand for 3D-printed bone will only increase in the coming years. By utilizing low-cost material, such as ABS in our study, and showing its biocompatibility and cellular response, the road to custom-created bone graft substitutes is easier and more affordable.

The trabecular patterning of the normal bone to create biomimetic structures, a unique feature compared to other approaches [13] was used in this study for two main reasons: to create a more conductive environment for bone matrix formation and, also, to mimic the structural support that trabeculae pattern gives to the bone. For instance, cancellous bone is the primary host of cellular regeneration; its built-in porosity leads to a broader surface area, which induces cells to inhabit in that surface more easily, hence, a better habitat for bone formation. Extracting the trabecular pattern from micro-CT scans and including it in the scaffold’s design allowed us to enhance the strength of the scaffolds in physiologic load-bearing directions, as seen in the results of our compression testing.

Culturing these scaffolds with osteoblasts leads to the production of mineralized matrix components that increase the stiffness and the strength of the scaffolds as well, which we have shown in our series of experiments. The type of osteogenic reagent used also showed differences, with CRFP treatment resulting in greater increases in compressive strength and modulus. The main goal is to promote cellular bone matrix propagation over inner and outer surfaces of scaffolds. This will constitute the biomimetic characteristic of these “frames” as they are hosting real cellular matrix, in addition to trabecular pattern already printed. We proposed that by allowing the cultured cells to mineralize the ABS scaffolds, the mechanical strength, particularly under compression stress, would be enhanced. Also, the biological performance of these scaffolds will be different compared to implants used nowadays as they will recreate a familiar environment where host cells will more likely grow and, therefore, allow for resorption and vascularization of the patient’s bone.

A few materials like mineralized collagen [14] and hydroxyapatite [15] allow osteogenic activity and are capable of guiding bone regeneration. However, these materials are very difficult to use in fabricating bony scaffolds, and the mechanical strength of this artificial bone is too low to provide effective support at load-bearing sites. After inducing mineralization over 3D-printed ABS scaffolds, it is also our interest to test the mechanical properties of these hybrids (plastic + organic). FDM process, like the majority of 3D printing procedures, builds the scaffolds layer by layer. This leads us to thinking about the anisotropy characteristic of our specimens. The introduction of the trabecular pattern on the scaffold’s design was also evaluated as a characteristic that would enhance the mechanical strength of the scaffolds. To do so, a scaffold design with no trabecular pattern, also called *flat* scaffold, was compared with the standard trabecular scaffold. Lastly, to assess the contribution of the extra cellular matrix of which the scaffolds were layered with, a comparison between non-treated scaffolds and treated scaffolds, i.e., after matrix formation, was conducted.

A limitation in this study is the use of ABS material to create scaffolds, as integrating these scaffolds into segmental bone defects and evaluating long-term sustainability will be a challenging task. Further experiments are being conducted to match the material properties of these scaffolds with those of the natural bone. Based on our results, we propose to create artificial bone using CRFP-coated 3D-printed scaffolds, seeded with stem cells to create a bone that will be tested for their anisotropy. The structural and functional integrity of the bone deposited artificial scaffolds will be evaluated in a mouse femoral segmental defect model in the next phase of the project. The ultimate goal of this project is to create artificial bone that is both biomimetic and biocompatible in humans.

## Conclusions

Currently, orthopedic and soft-tissue injuries account for ~ 75% of all injuries. Unfortunately, advances in management of orthopedic injuries have not kept pace with their increased incidence, resulting in unacceptably high levels of medical discharges and life-long disability as well as tremendous financial burdens associated with long-term care and rehabilitation. The experiments outlined in this work tested whether the artificial scaffolds, seeded with bone-producing cells and treated with osteo-inductive agents, are mechanically stronger. Upon mineralization, the cell-seeded trabecular implants treated with osteo-inductive agents and pretreated with CRFP showed a significant increase in the compressive modulus, suggesting that these scaffolds are biocompatible. Testing with different biocompatible materials and the results obtained from this work will lead to creating 3D structures that can be used in the replacement of not only the bone segments but the entire bones, particularly the wrist and ankle bones, as fractures or deformities of these irregular small bones play a critical role in the morbidity of patients.
