# Correction to: Biomechanical properties of 3D-printed bone scaffolds are improved by treatment with CRFP

**DOI:** 10.1186/s13018-018-0741-1

**Published:** 2018-02-19

**Authors:** Carlos G. Helguero, Vamiq M. Mustahsan, Sunjit Parmar, Sahana Pentyala, John L. Pfail, Imin Kao, David E. Komatsu, Srinivas Pentyala

**Affiliations:** 1 Department of Mechanical Engineering, Stony Brook University, Stony Brook, NY USA; 2 Department of Orthopedics, Stony Brook Medical Center, Stony Brook, NY USA; 3 Department of Anesthesiology, Stony Brook Medical Center, Stony Brook, NY USA; 4 Facultad de Ingeniería en Mecánica y Ciencias de la Producción, Escuela Superior Politécnica del Litoral, ESPOL, Guayaquil, Ecuador

## Correction to: J Orthop Surg Res (2017) 12: 195. https://doi.org/10.1186/s13018-017-0700-2

In the original publication of this article [1] there was an error in one of the author names. In this publication the correct and incorrect name are indicated.

Originally the author name has been published as:

• John P. Pfail

The correct name is as followed:

• John L. Pfail

The original publication has been corrected.
